# Anharmonicity and isomorphic phase transition: a multi-temperature X-ray single-crystal and powder diffraction study of 1-(2′-aminophenyl)-2-methyl-4-nitroimidazole

**DOI:** 10.1107/S2052252514002838

**Published:** 2014-02-28

**Authors:** Agnieszka Poulain, Emmanuel Wenger, Pierrick Durand, Katarzyna N. Jarzembska, Radosław Kamiński, Pierre Fertey, Maciej Kubicki, Claude Lecomte

**Affiliations:** a CRM^2^, Jean Barriol Institute, CNRS UMR 7036, University of Lorraine, BP 70239, Boulevard des Aiguillettes, 54506 Vandoeuvre-lès-Nancy, France; b Faculty of Chemistry, Adam Mickiewicz University, Umultowska 89B, 61-614 Poznań, Poland; c Department of Chemistry, University of Warsaw, Pastuera 1, 02-093 Warszawa, Poland

**Keywords:** anharmonicity, isomorphic phase transition, experimental charge density, X-ray closed-circuit helium cryostat, Hansen–Coppens model, multiple-temperature powder diffraction

## Abstract

The harmonic model of atomic nuclear motions is usually enough for multipole modelling of high-resolution X-ray diffraction data; however, in some molecular crystals, such as 1-(2′-aminophenyl)-2-methyl-4-nitro-1*H*-imidazole [Paul, Kubicki, Jelsch *et al.* (2011 ▶). *Acta Cryst.* B**67**, 365–378], it may not be sufficient for a correct description of the charge-density distribution. Multipole refinement using harmonic atom vibrations does not lead to the best electron density model in this case and the so-called ‘shashlik-like’ pattern of positive and negative residual electron density peaks is observed in the vicinity of some atoms. This slight disorder, which cannot be modelled by split atoms, was solved using third-order anharmonic nuclear motion (ANM) parameters. Multipole refinement of the experimental high-resolution X-ray diffraction data of 1-(2′-aminophenyl)-2-methyl-4-nitro-1*H*-imidazole at three different temperatures (10, 35 and 70 K) and a series of powder diffraction experiments (20 ≤ *T* ≤ 300 K) were performed to relate this anharmonicity observed for several light atoms (N atoms of amino and nitro groups, and O atoms of nitro groups) to an isomorphic phase transition reflected by a change in the *b* cell parameter around 65 K. The observed disorder may result from the coexistence of domains of two phases over a large temperature range, as shown by low-temperature powder diffraction.

## Abstract 2

Multi-temperature single-crystal and powder diffraction experiments on 1-(2′-aminophenyl)-2-methyl-4-nitroimidazole show that this crystal undergoes an isomorphic phase transition with the coexistence of two phase domains over a wide temperature range. The anharmonic approach was the only way to model the resulting disorder.

## 1. Introduction   

When using accurate ultra-high-resolution X-ray diffraction data, the most commonly used harmonic model of the atomic nuclear motions may not be sufficient for some molecular crystals, even for lighter atoms. Therefore, multipole refinement without modelling anharmonic nuclear motions (ANMs) does not lead to the best electron density (ED) model, as revealed by peaks and holes in residual maps; these peaks arranged in a ‘shashlik-like’ pattern in the vicinity of the anharmonic atoms are an indicator of third-order ANMs (Herbst-Irmer *et al.*, 2013 ▶; Meindl *et al.*, 2010 ▶) and can be modelled by introducing Gram–Charlier or cumulant expansions (Johnson & Levy, 1974 ▶).

Despite the fact that ANMs have been previously discussed in the literature (*e.g.* Kuhs, 1988 ▶, 1992 ▶), their reliable separation from the static charge-density distribution parameters, disorder or librations was questioned (Mallinson *et al.*, 1988 ▶; Restori & Schwarzenbach, 1996 ▶). Although Iversen *et al.* (1999 ▶) distinguished anharmonic nuclear motions from static electron density features in a thorium complex structure using extremely high-resolution (1.7 Å^−1^) data from two very low-temperature experiments (at 9 and 27 K), Henn *et al.* (2010 ▶) were able to separate both contributions for lighter atoms (namely P atoms) at lower resolution (1.15 Å^−1^) at 100 K. Birkedal *et al.* (2004 ▶) successfully refined the multipolar electron density of urea, while Scheins *et al.* (2010 ▶) showed that ANMs are necessary for the correct description of the charge density of a Zn atom. Finally, Zhurov *et al.* (2011 ▶) showed that neglecting ANMs in the case of hexahydro-1,3,5-trinitro-1,3,5-triazine (RDX) results in unrealistic charge-density deformation and Laplacian maps in the region of the nitro group. For a similar compound, 1,3,5,7-tetranitro-1,3,5,7-tetraazacyclooctane (HMX), which has a slightly more compact crystal structure, the refined ANM parameters were statistically significant, however, their effect on the resulting charge-density deformation and Laplacian maps was rather negligible.

The effects related to ANMs are visible only at high-resolution data and the values representing the corresponding refined Gram–Charlier coefficients are often hardly statistically significant. Correlatively, the agreement factors do not improve noticeably upon the introduction of these new parameters. Nevertheless, such a physical model considerably reduces residual peak heights (Paul, Kubicki, Jelsch *et al.*, 2011 ▶; see Figs. 4 and 5 therein). To avoid possible correlations between ANMs and the remaining ED parameters, the former ones should be refined first against high-resolution data and then by a joint refinement of both anharmonic and electron density parameters in the subsequent refinement steps (Mallinson *et al.*, 1988 ▶).

Standard resolution crystal structures of numerous 4-nitroimidazole derivatives have been investigated in our laboratories, with special attention paid to the weak intermolecular interactions present in these molecular crystals (Kubicki *et al.*, 2001 ▶; Kubicki, 2004*a* ▶,*b* ▶; Kubicki & Wagner, 2007 ▶, 2008 ▶; Wagner *et al.*, 2007 ▶; Wagner & Kubicki, 2007 ▶). Further investigations of the high-resolution diffraction data using the Hansen–Coppens model (Hansen & Coppens, 1978 ▶) and quantum theory of atoms in molecules (QTAIM; Bader, 1994 ▶) topological analysis were performed for 1-phenyl-4-nitroimidazole (Kubicki *et al.*, 2002 ▶), 1-(2′-aminophenyl)-2-methyl-4-nitroimidazole (Paul, Kubicki, Jelsch *et al.*, 2011 ▶), 2-methyl-4-nitro-1-phenyl-1*H*-imidazole-5-carbonitrile (Poulain-Paul *et al.*, 2012 ▶; Paul, Kubicki, Kubas *et al.*, 2011 ▶) and for the solid solution of 1-(4′-chlorophenyl)-2-methyl-4-nitro-1*H*-imidazole-5-carbonitrile (97.5%) with 5-bromo-1-(4′-chlorophenyl)-2-methyl-4-nitro-1*H*-imidazole (2.5%; Poulain *et al.*, 2014 ▶).

After high-resolution crystal structure determination and multipolar refinement of 1-(2′-aminophenyl)-2-methyl-4-nitroimidazole, **1** [Chem scheme1], at 100 K (Paul, Kubicki, Jelsch *et al.*, 2011 ▶), unexpected high residual-density peaks arranged in a ‘shashlik-like’ pattern appeared at high-order residual maps (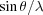 ≥ 0.7 Å^−1^) in the planes bisecting the amino groups of two symmetry-independent molecules, and a distorted static deformation density was observed for one of the nitro groups involved in the weaker hydrogen bonds. Thus, third-order ANMs were used to model the two fragments of the molecules (a split-atom refinement did not succeed). Such a procedure resulted in virtually featureless residual electron density maps and symmetrical arrangement of the static electron density of the NO_2_ fragment.
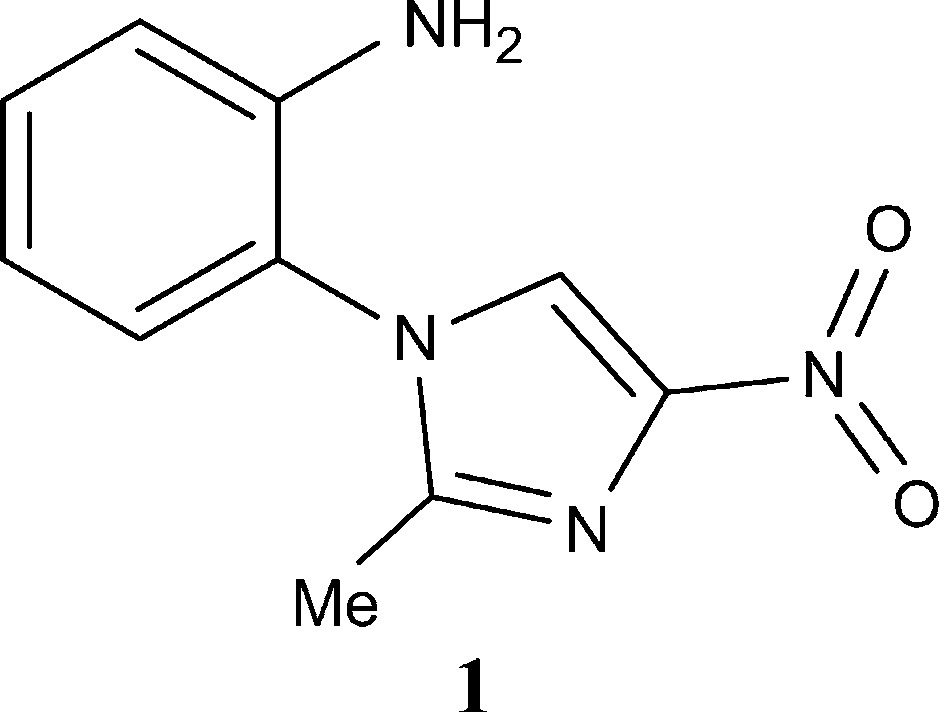


In the next step powder diffraction experiments at different temperatures (20 ≤ *T* ≤ 300 K) were performed. The data collected revealed an isomorphic phase transition (see for example Bendeif *et al.*, 2009 ▶) as reflected by an abrupt change of the *b* unit-cell parameter around 65 K. Forbidden reflections in 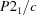 did not appear, which suggests that the space group was conserved.

The aim of this paper is an attempt to relate this anharmonic refinement to the isomorphic phase transition by analysing several additional X-ray single-crystal diffraction experiments performed for **1**, including a high-resolution full data collection at 10 K on an Agilent Technologies SuperNova diffractometer, accurate full data collections at 35 and 70 K using the homemade mini-goniometer system implemented on an Orange top-loading cryostat on the CRM^2^ Bruker AXS APEX II diffractometer (Fertey *et al.*, 2007 ▶), and temperature-dependent unit-cell parameter determination from powder diffraction patterns collected on a Panalytical X’Pert Pro diffractometer. As careful crystal structure and topological analyses of the electron density have already been performed for the title compound (Paul, Kubicki, Jelsch *et al.*, 2011 ▶; Kubicki & Wagner, 2008 ▶), they are not repeated in this paper.

## 2. Materials and methods   

### 2.1. Experimental details of X-ray single-crystal diffraction measurements   

A yellowish transparent cube-shaped crystal (0.20 × 0.17 × 0.13 mm) was chosen for data collection at 10 K on an Agilent Technologies SuperNova four-circle diffractometer equipped with a CCD detector. The temperature was controlled with an Oxford Cryosystems cooling device. A total of 2970 frames were collected in 35 runs to obtain the high redundancy data and 32 additional reference frames were measured to verify the stability of the crystal. Diffraction data up to 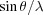 = 1.10 Å^−1^ were collected using the ω-scan method with a rotation width of 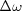 = 1°. Different exposure times were chosen depending on the 2θ settings of the detector: 5 s for 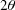 = 1.25°, and 20 s for 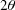 = −65.45 and 67.95°, with a 55 mm crystal-to-detector distance. Details of the data collection and crystallographic statistics are collected in Table 1 ▶.

Another yellowish crystal (0.12 × 0.14 × 0.15 mm) from the same crystallization batch was chosen for the 35 and 70 K measurements on a CRM^2^ Nonius Kappa CCD diffractometer equipped with a homemade universal low-temperature mini-goniometer, helium top-loading Orange cryostat (Fertey *et al.*, 2007 ▶). A total of 4074 (35 K) and 8240 (70 K) frames were collected in 8 (35 K) and 25 runs (70 K). Diffraction data up to 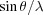 = 0.90 Å^−1^ (35 K) and 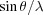 = 1.20 Å^−1^ (70 K) – the lower resolution for the 35 K measurement resulted from time and liquid helium restrictions – were collected using ω- and ϕ-scan methods with 0.25° rotation widths, the χ angle being fixed at 43.37° and the crystal-to-detector distance at 40 mm. Two 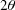 positions (−30 and −90°) were used to collect all the reflections with exposure times of 3 and 60 s for the 35 K data, and 10 s and 60 s for the 70 K data. Details of the data collections and measurement statistics are given in Table 1 ▶. Despite some geometrical constraints due to the cryostat orientation, the completeness of the data is very close to 100%, and the internal agreement factors are very good compared with typical charge-density quality X-ray data.

Unit-cell parameter determination, integration of the reflection intensities, data reduction and Lorentz–polarization corrections were performed using *CrysAlis PRO* (Agilent Technologies, 2013 ▶) for the 10 K data, and *APEX2* (Bruker, 2012 ▶) for the 35 and 70 K data. An analytical numerical absorption correction using a multi-faced crystal model (Clark & Reid, 1995 ▶) was applied to the 10 K data, while a multi-scan absorption correction (Blessing, 1995 ▶) was applied to the 35 and 70 K data. Data sorting, scaling and merging of reflections were performed with *SORTAV* (Blessing, 1997 ▶, 1989 ▶, 1987 ▶) for all three datasets.

As shown in Table 1 ▶, all the multipolar models (10, 35 and 70 K) converge to very good *R* factors. This shows the possibility of collecting accurate charge density data using the mini-goniometer and cryostat system (Fertey *et al.*, 2007 ▶), *i.e.* performing very low-temperature high-resolution accurate X-ray data collections with very small helium consumption. One of the problems not yet resolved for the mini-goniometer data is the precision of the cell parameters (Table 1 ▶) possibly due to the difficulty in centering the crystal inside the cryostat, and to the anisotropy of the data collection needed to avoid possible collisions; as shown below, this has some consequences on the quality of the bond distances and angles.

### 2.2. Powder diffraction measurements (PXRD)   

All PXRD measurements were performed using a Panalytical X’Pert Pro diffractometer equipped with a Cu tube, a Ge(111) incident-beam monochromator (λ = 1.5406 Å) and an X’Celerator detector. Temperature-controlled diffractograms were collected on cooling with an Oxford Cryosystems cryostat (Phenix) from 300 to 125 K (under vacuum, cooling rate 6 K min^−1^; 25 K increments; temperature stabilization: 5 min), then from 120 to 15 K (under vacuum, cooling rate 6 K min^−1^, 5 K increments, temperature stabilization: 5 min). Temperature-controlled diffractograms were collected on heating from 20 to 120 K with the same cryostat and then from 125 to 300 K under the same conditions.

Data collection was carried out in the scattering angle range θ = 5–50° with a 0.0167° step over 4 h. The program *GSAS/EXGUI* (Toby, 2001 ▶; Larson & Von Dreele, 1994 ▶) was used for the Le Bail extraction in space group 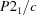. Owing to the complexity of the structure and since powder X-ray diffraction (PXRD) is less sensitive than single-crystal measurements, single-crystal atomic parameters were used as the structural model. Only the cell dimensions, parameters of the pseudo-Voigt profile shape function and the zero shift were refined.

### 2.3. Structure determination and refinement   

**Figure 1:**
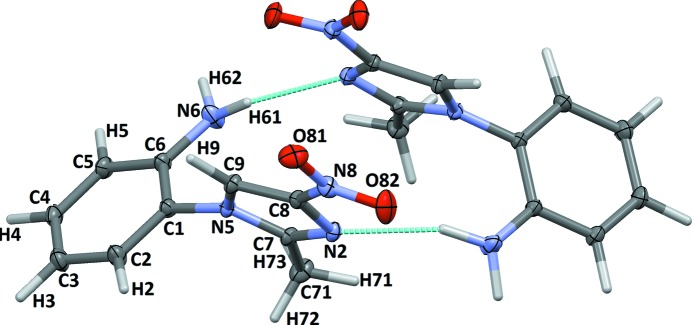
Anisotropic ellipsoid representation of the two symmetry-independent molecules of **1** with atom-labelling scheme. Ellipsoids are drawn at the 50% probability level, H atoms are depicted as capped sticks (*MERCURY*; Macrae *et al.*, 2008 ▶). The labels of the second molecule are ordered in the same way and marked with an *A* (*e.g.* C1*A*, N1*A* *etc.*). The strongest interactions are indicated by turquoise dashed lines.

Crystal structures of **1** for the three datasets (10, 35 and 70 K) were solved using *SIR92* (Altomare *et al.*, 1993 ▶) and first refined with *SHELXL* (Sheldrick, 2008 ▶) applying the independent atom model (IAM), with isotropic and anisotropic treatment of H and non-H atoms, respectively. Geometry constraints (C_Ar_—H = 1.083 Å; C_Me_—H = 1.059 Å; N—H = 1.009 Å), atomic thermal motion parameters (initial values of 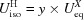; *y* = 1.2 for Ar and NH_2_ groups; *y* = 1.5 for Me group) were initially imposed on H atoms to preserve the physical meaningfulness of the models. Fig. 1 ▶ shows the two symmetry-independent molecules of **1** with labelling scheme (see Paul, Kubicki, Jelsch *et al.*, 2011 ▶, for more details).

Subsequently the multipolar refinement strategy previously presented was applied, with restraints on symmetry and chemical equivalency defined as optimal from 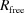-factor calculations (see Paul, Kubicki, Jelsch *et al.*, 2011 ▶, and references therein). The main points of the refinement strategy were the following: (*a*) scale factor refined continuously with all parameters; (*b*) anharmonicity parameters refined against the high-order data (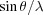 ≥ 0.7 Å^−1^; only deemed necessary for the 70 K data); (*c*) thermal motion and positional parameters for non-H atoms against high-order data alternatively with H-atom coordinates and distances constrained to standard neutron values (Allen & Bruno, 2010 ▶); (*d*) refinement of multipolar parameters followed by valence populations (constraints imposed on chemically equivalent atoms in a similar environment) and then both together; (*e*) κ parameters for non-H atoms (constraints imposed on chemically equivalent atoms in similar environment); (*f*) points (*d*) and (*e*) performed until convergence is achieved; (*g*) anharmonicity parameters (only for 70 K data) alternatively with thermal motion and positional parameters for all atoms against all data (H atoms still constrained); (*h*) valence and multipole populations alternatively with κ for non-H atoms and positional parameters plus thermal motion; (*i*) anharmonicity parameters (only for 70 K data); constraints on valence and multipole populations together with κ, 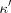 coefficients changed into restraints at the 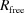 level; (*j*) 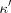 non-H atoms alternatively with valence and multipole populations; (*k*) κ of H atoms; (*l*) points (*h*) and (*i*) alternatively; (*m*) 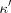 for H atoms; (*n*) point (*l*) repeated; (*n*) *SHADE* estimation of the thermal motion of H atoms (Madsen, 2006 ▶; Madsen *et al.*, 2013 ▶); (*o*) valence and multipole populations alternatively with κ H atoms, κ non-H atoms, coordinates and thermal motion; (*p*) point (*j*) repeated; (*q*) point (*h*) repeated; successive refinement of κ non-H, κ H-atoms, 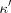 non-H, 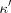 H atoms; (*r*) final simultaneous refinement of all parameters.

As mentioned above, only the 70 K data required the third-order anharmonic corrections (Kuhs, 1992 ▶; Sørensen *et al.*, 2003 ▶) for a correct modelling of three atoms of one NO_2_ group (N81, O81 and O82) and two amino N atoms (N6 and N6*A*) in order to reduce the typical ‘shashlik-like’ pattern usually found at high-order residual density maps. Stronger interactions, in which the second nitro group (N81*A*, O81*A* and O82*A*) is involved, seem to restrict vibrations and therefore an harmonic model was sufficient.

## 3. Results and discussion   

### 3.1. Powder diffraction data   

**Figure 2:**
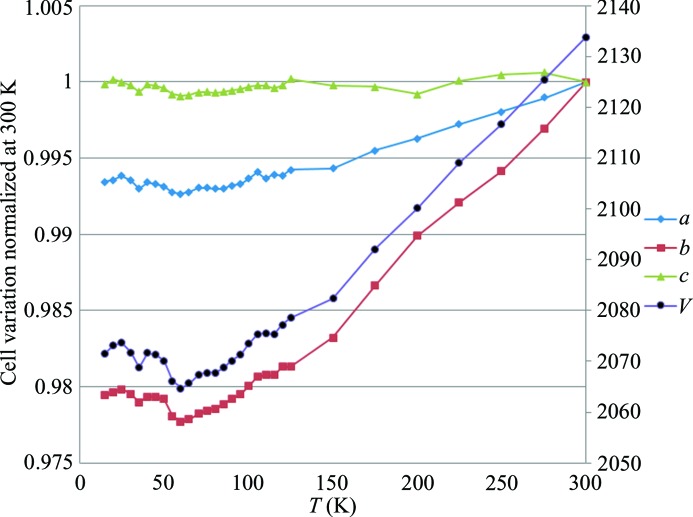
Unit-cell parameter variation with temperature decrease from 300 to 20 K normalized to 300 K.

Along with the temperature decrease from 300 to 100 K, a linear evolution of the unit-cell volume is observed reaching a minimum at ∼ 60–65 K (Fig. 2 ▶), followed by a slight volume increase from 50 to 20 K. The *b* parameter decreases linearly from room temperature (RT) to 60 K with the temperature (*T*) (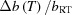 = −3 × 10^−5^ *T* + 0.99) and then increases for *T* < 60 K (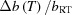 = 2 × 10^5^ *T* + 0.99), in agreement with previous findings (Paul, Kubicki, Jelsch *et al.*, 2011 ▶). The *c* parameter remains almost constant, as already noted by Paul, Kubicki, Jelsch * et al.* (2011 ▶). Contrary to the observation of Bendeif *et al.* (2009 ▶) no hysteresis phenomenon was found or if it exists the temperature difference is smaller than 5 K.

**Figure 3:**
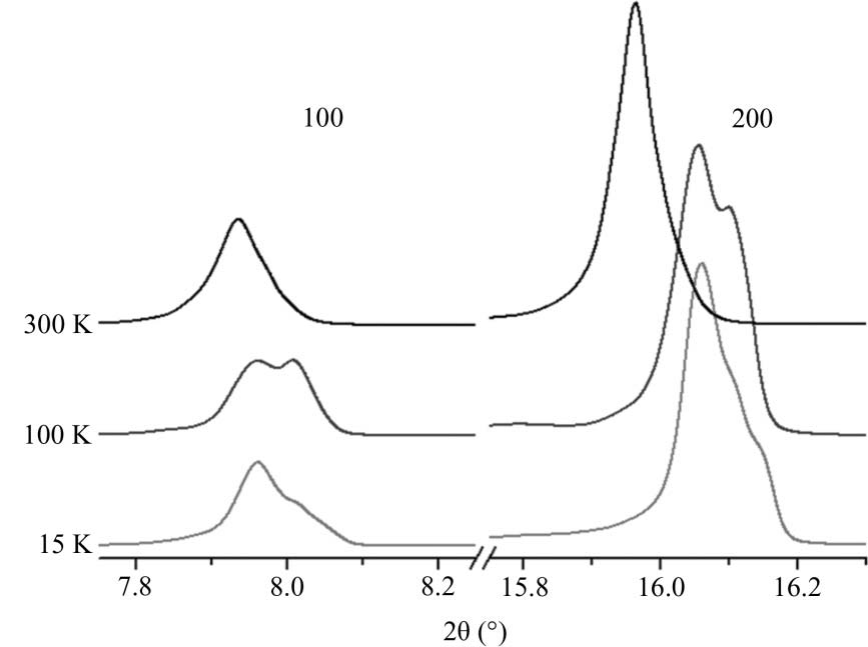
Splitting of the 100 and 200 diffraction peaks with temperature increase.

When increasing *T* in the range 15–100 K, a splitting of the 100 and 200 reflections appears, which may suggest a second-order phase transition (Fig. 3 ▶). The phenomenon is more pronounced at 100 K, while at 300 K the diffraction peaks are practically symmetrical. Such a splitting is not visible on the 020 reflection due to its very small intensity (Fig. S2). Two crystal phases seem to coexist along a large temperature range. This can explain the observed disorder at 100 K, which was solved using anharmonic atom treatment.

### 3.2. Charge-density distribution modelling   

According to our previous findings (Paul, Kubicki, Jelsch *et al.*, 2011 ▶) for the 100 K data, the largest residual peaks in the residual density Fourier maps (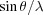 ≤ 0.9 Å^−1^) lie in the planes bisecting the H61—N6—H62 moiety, at a distance of *ca* 0.5 Å from the N atoms (0.37 e Å^−3^ for N6*A*, and 0.28 e Å^−3^ for N6 atoms). They disappear at a resolution of 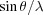 ≤ 0.7 Å^−1^ and therefore cannot be interpreted as missing H atoms, because they only appear when high-order reflections are included, while H atoms scatter at very low 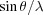. The refined third-order anharmonic parameters are statistically not significant, but reduce substantially the residual peak heights.

The first important result of this report is that the 10 and 35 K data do not need any anharmonic motion modelling (ANM) of both amino and nitro groups, whereas ANM refinement is still necessary at 70 K as peaks and holes in the ‘shashlik-like’ pattern appear close to the N6 atom: +0.42 (6) and −0.32 (6) e Å^−3^ [compared with +0.56 (5) and −0.27 (5) e Å^−3^ for the 100 K data (Paul, Kubicki, Jelsch *et al.*, 2011 ▶)]. The lower resolution of the 35 K (0.9 Å^−1^) dataset compared with the 10 K (1.1 Å^−1^) and 70 K (1.2 Å^−1^) ones does not affect the detectability of the ‘shashlik-like’ pattern, since such a distortion is already observed at 100 K at 0.9 Å^−1^ cut-off (Paul, Kubicki, Jelsch *et al.*, 2011 ▶). Moreover, the 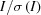 cut-off at 35 K was reduced to 1.25 compared with 2.0 for 10 and 70 K in order to improve the data-to-parameter ratio.

**Figure 4:**
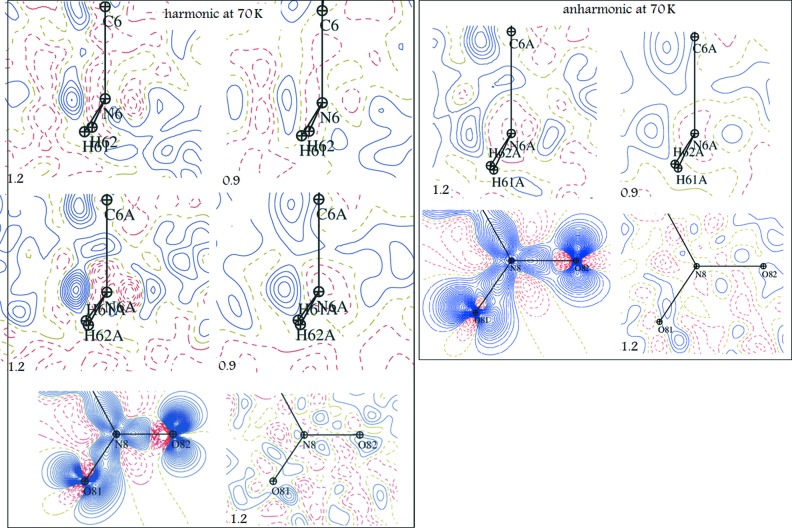
Residual and static electron density maps at 70 K after multipole refinement in the planes bisecting NH_2_ and NO_2_ groups (cutoff 1.2 and 0.9 Å resolution) *neglecting* (left panel) or *including* (right panel) ANMs; contours set to 0.05 e Å^−3^, blue dashed lines – negative contours, red solid lines – positive contours; 1.2 and 0.9 values indicate the resolution (Å^−1^).

Fig. 4 ▶ gives residual density maps obtained after harmonic (left panel) and anharmonic (right panel) treatment of the 70 K data. Similar to the 100 K data, residual peaks at 70 K are more pronounced for one of the two amino groups (N6*A*) and mostly at higher resolution (1.2 Å^−1^). The residual peaks at 100 K are slightly higher than those observed for the 70 K data. Application of the ANMs of third-order significantly reduced the positive and negative residual electron density peaks and restored the expected valence-density arrangement around O atoms in the NO_2_ group.

Comparison of the third-order ANM parameters for the 100 and 70 K data is given in Table 2 ▶ for 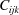 above the 3σ criterion. There is a general trend that the significant parameters at 100 K drop considerably at 70 K (*e.g.* 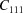 for N6 and N6*A* atoms). However, surprisingly, some parameters seem to be significant only at 70 K (*e.g.* 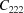, 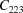 and 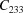 for N6*A*).

The quality of the four (10, 35, 70 and 100 K) data refinements is comparable, with insignificant differences between the corresponding agreement factors (Table 1 ▶): 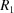 = 0.029–0.032, 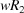 = 0.025–0.028 and *S* (goodness-of-fit) = 0.90 (10 K)–1.07 (100 K), and 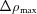 (from +0.25 e Å^−3^ to +0.32 e Å^−3^), 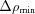 (from −0.22 e Å^−3^ to −0.34 e Å^−3^), which in fact depends on the data collection resolution (lowest for 35 K data).

**Figure 5:**
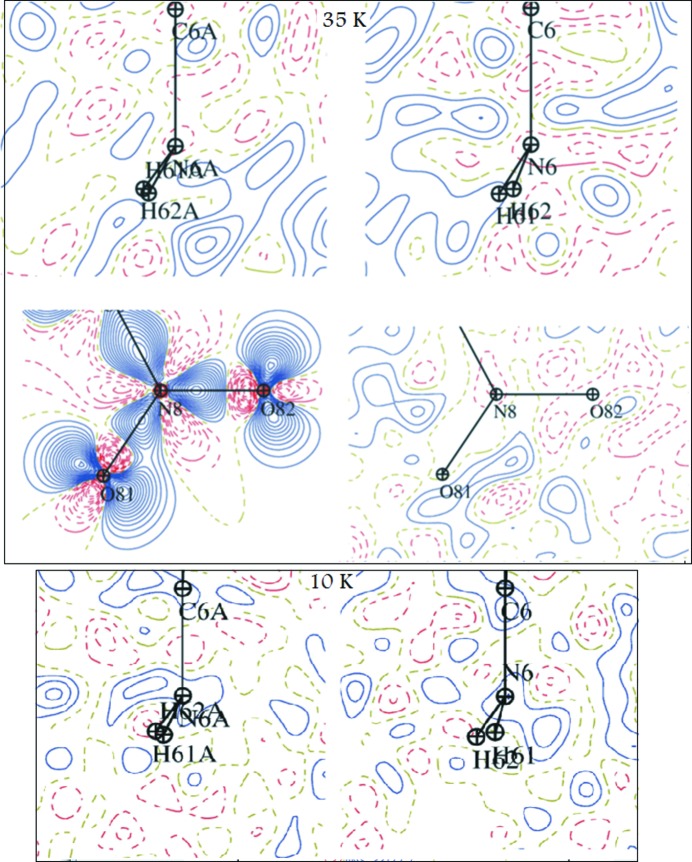
Residual electron density and static deformation maps after *harmonic* modelling of 35 and 10 K data drawn in the planes bisecting both amino groups and one nitro group prone to geometrical distortion; contours set at 0.05 e Å^−3^, blue solid lines – positive contours, red dashed lines – negative contours, 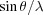 ≤ 0.9 Å^−1^.

In conclusion, diffraction experiments at 35 and 10 K did not require any special anharmonic treatment, as the harmonic approximation is sufficient for all the atoms concerned (Fig. 5 ▶). It is in line with the isomorphic phase transition, which occurs around 60 K. ANH modelling of the 70 and 100 K data enables modelling of the residual density accounting for the disorder which may be due to the co­existence of both LT and HT crystal phases existing in this temperature range.

### 3.3. Electron density model validation *via* topological analysis of the covalent bonds   

In order to compare and validate the model correctness at different temperatures (10, 35, 70 and 100 K) the covalent bond critical points (CPs) of the aryl ring (that should be unchanged and prove consistency of these four data treatments), together with those of the anharmonic fragments, are collected in Table S1. In general, the distance between the two involved atoms is ∼ 0.01 Å longer for the 70 K structure, but this lengthening is not significant enough to be reflected in the respective distances to the critical points and, as seen below, is a result of a less accurate estimation of the cell parameters derived from the mini-goniometer data. For the C—C bonds of the aryl ring the total electron density value differences for a given bond are ≤ 0.1 e Å^−3^, about 2σ, while the Laplacian values are systematically higher for the 70–100 K data, but within the usually accepted estimated error (up to 4.0 e Å^−5^).

For bonds involving the anharmonic atoms the total density at CP is on average larger for the datasets, which were corrected for anharmonic treatment (maximal change 0.2 e Å^−3^ for the N8—O81 bond), as well as the Laplacian values ≃ 4–6 e Å^−5^) for all the 70–100 K bonds, except N8—O81.

**Figure 6:**
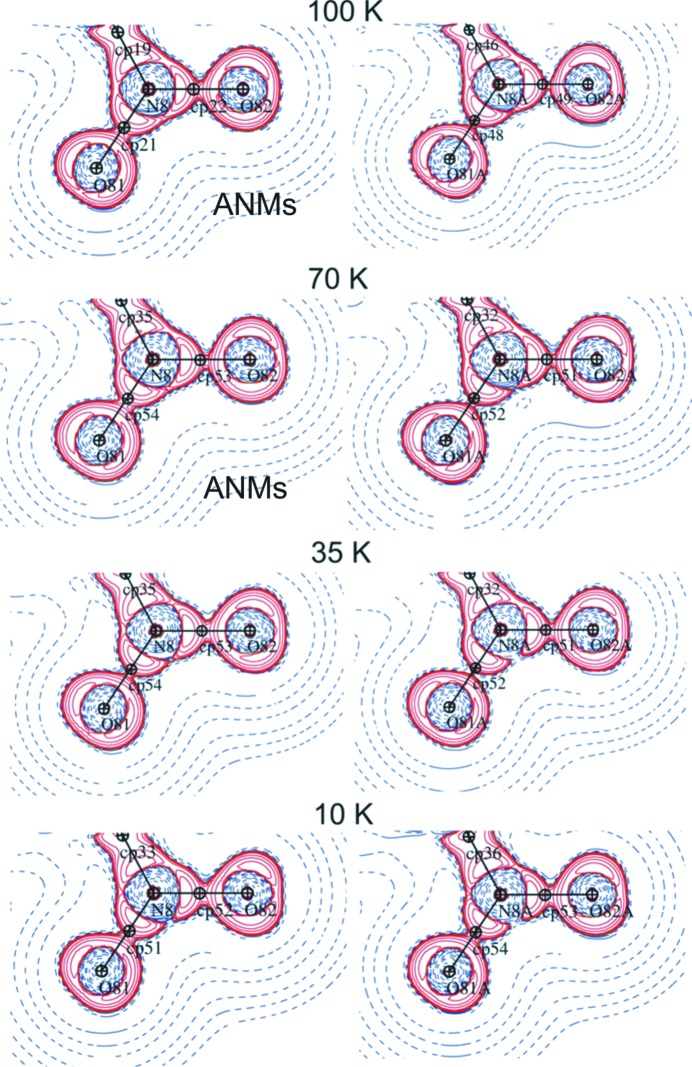
Laplacian of the total electron density maps at 100, 70, 35 and 10 K for the two NO_2_ groups; anharmonic treatment indicated by ANMs marked; logarithmic contours; blue dashed lines – positive contours, red solid lines − negative contours.

Contrary to Zhurov *et al.* (2011 ▶) the Laplacian maps (Fig. 6 ▶) of the nitro group calculated within the harmonic approximation (not shown here) are indistinguishable from those correctly modelled, which suggests a lower anharmonicity/disorder in **1**.

For the three critical points characterizing the strongest intermolecular interactions where the NO_2_ groups are involved, the topological data at different temperatures are collected in Table 3 ▶. All electron density values decrease when the temperature increases, while the Laplacian values fluctuate rather than show a visible trend. Nevertheless, all these changes are insignificant at the 3σ level, as expected on the basis of constant intermolecular distances [for example, the O81*A*⋯H62*A* distance equals 2.028 (10) Å].

In a recent review, Kamiński *et al.* (2014 ▶) investigated structural parameters and charge-density properties in a series of 100 K high-resolution datasets of α-oxalic acid dehydrate, which reveals that electron density and Laplacian values at corresponding CPs for this unique crystal structure vary over a small range, even at the same temperature. The standard deviations for the total electron density and Laplacian for covalent bonds and intermolecular bonds vary between 0.03–0.06 e Å^−3^, 1–7 e Å^−5^ and 0.001–0.03 e Å^−3^, 1–6 e Å^−5^, respectively, which confirms our above conclusion that changes in **1** are statistically insignificant.

### 3.4. Accuracy of the bond lengths obtained from the mini-goniometer data   

As already shown recently (Jarzembska *et al.*, 2013 ▶), it is difficult to obtain an accurate orientation matrix with the mini-goniometer setup, leading to slightly different cell parameters compared with those obtained from powder diffraction data, which consequently affects the precision of the bond distances. Recalculation of the aryl ring C—C bond lengths for the 35–100 K data, using the unit-cell parameters obtained from the powder diffraction experiment (second row of Table 4 ▶), brings a much better agreement (Table S1 *versus* Table 5 ▶). The maximal difference in the 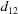 value between 35 and 100 K is 0.008 Å and a clear trend is found: 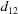 (35 K) > 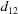 (70 K) > 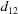 (100 K). This behaviour has been known for a few decades (see for example Busing & Levy, 1964 ▶; Scheringer, 1980 ▶; Destro & Merati, 1995 ▶), and results from the higher degree of precision in determining molecular geometry at lower temperatures.

## 4. Conclusions   

The aim of this study was to show the link between anharmonicity and isomorphic phase transition of a molecular crystal. We have shown that ANM corrections improve the charge-density model above the phase transition temperature, whereas a simple harmonic model is sufficient below the transition temperature. Softening of the anharmonicity is therefore connected with the transition mechanism. As shown from powder diffraction data the nature of the phase transition seems to be second order with a coexistence of both phases over a large temperature range (40–50 K). As the atomic structures of both phases are extremely similar, a split-atom model cannot take into account the disorder observed on the residual maps which was accounted for using a third-order anharmonic treatment. Such an interpretation however needs more experiments on other molecular crystals to be considered as a general rule.

## Supplementary Material

Crystal structure: contains datablock(s) I_10K, I_35K, I_70K. DOI: 10.1107/S2052252514002838/gq5001sup1.cif

Structure factors: contains datablock(s) I_10K. DOI: 10.1107/S2052252514002838/gq5001I_10Ksup2.fcf

Structure factors: contains datablock(s) . DOI: 10.1107/S2052252514002838/gq5001I_35Ksup3.fcf

Structure factors: contains datablock(s) I_70K. DOI: 10.1107/S2052252514002838/gq5001I_70Ksup4.fcf

Rietveld powder data: contains datablock(s) Icooling_15K. DOI: 10.1107/S2052252514002838/gq5001Icooling_15Ksup5.rtv

Rietveld powder data: contains datablock(s) Icooling_35K. DOI: 10.1107/S2052252514002838/gq5001Icooling_35Ksup6.rtv

Rietveld powder data: contains datablock(s) Icooling_70K. DOI: 10.1107/S2052252514002838/gq5001Icooling_70Ksup7.rtv

Rietveld powder data: contains datablock(s) Icooling_100K. DOI: 10.1107/S2052252514002838/gq5001Icooling_100Ksup8.rtv

Rietveld powder data: contains datablock(s) Iheating_15K. DOI: 10.1107/S2052252514002838/gq5001Iheating_15Ksup9.rtv

Rietveld powder data: contains datablock(s) Iheating_35K. DOI: 10.1107/S2052252514002838/gq5001Iheating_35Ksup10.rtv

Rietveld powder data: contains datablock(s) Iheating_70K. DOI: 10.1107/S2052252514002838/gq5001Iheating_70Ksup11.rtv

Rietveld powder data: contains datablock(s) Iheating_100K. DOI: 10.1107/S2052252514002838/gq5001Iheating_100Ksup12.rtv

Supporting figures and table. DOI: 10.1107/S2052252514002838/gq5001sup13.pdf

CCDC references: 967290, 967291, 967292

## Figures and Tables

**Table 1:** Experimental details for single-crystal measurements at 10, 35 and 70 K – all refinement parameters are given for the multipole model

Crystal data			
Chemical formula	C_10_H_10_N_4_O_2_		
*M*_r_	436.4		
Temperature (K)	10	35	70
Wavelength (Å)	0.71073, graphite-monochromated		
Crystal system, space group	Monoclinic, *P*2_1_/*c*		
*a*, *b*, *c* (Å)	11.0104 (3), 10.0398 (2), 18.6040 (4)	10.9784 (14), 10.0056 (13), 18.488 (3)	11.0470 (12), 10.1293 (11), 18.652 (2)
β (°)	97.320 (2)	97.223 (4)	97.223 (3)
*V* (*Å^3^*)	2039.77 (8)	2014.7 (5)	2070.6 (4)
*Z*	8		
*D_x_* (g cm^−3^)	1.42	1.44	1.40
*F*_000_	912		
Absorption coefficient (mm^−1^)	0.104	0.105	0.102
Crystal to detector distance (mm)	55	40	40
			
Data collection			
Absorption correction	Analytical	Multi-scan	Multi-scan
*T*_min_, *T*_max_	0.983, 0.989	0.915, 1.105	0.932, 1.028
Crystal size (mm)	0.20 × 0.17 × 0.13	0.12 × 0.14 × 0.15	0.12 × 0.14 × 0.15
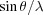 range (Å^−1^)	0.07–1.10	0.07–0.90	0.07–1.20
Limiting indices	−24 → *h* → 25, −22 → *k* → 22, −41 → *l* → 41	−19 → *h* → 16, −15 → *k* → 17, −32 → *l* → 32	−23 → *h* → 25, −23 → *k* → 22, −44 → *l* → 44
Reflections collected, unique, unique with σ cut-off	305 420, 22 731, 15 217 [*I* > 2σ(*I*)]	41 665, 11 032, 9475 [*I* > 1.25σ(*I*)]	121 651, 26 563, 17 738 [*I* > 2σ(*I*)]
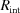	0.059	0.065	0.087
Data completeness (%)	100	96.7	88.5
No. of parameters	945	945	995†
			
Refinement			
Weighting scheme	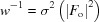		
Goodness of fit on *F* ^2^	0.90	0.92	0.95
Final *R*(*F*) indices‡	*R_1_* = 0.032, *wR_2_* = 0.028	*R*_1_ = 0.029, *wR*_2_ = 0.028	*R*_1_ = 0.029, *wR*_2_ = 0.028
Δρ_max_, Δρ_min_ (e Å^−3^)	0.32 (6), −0.34 (6)	0.25 (6), −0.30 (6)	0.29 (6), −0.27 (6)

† Different number of refined parameters due to additional ANMs required only at 70 K.

‡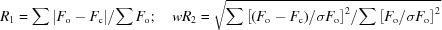.

**Table 2:** Anharmonic nuclear motion parameters greater than 3σ for the 100 K and 70 K data

100 K										
*C*_111_	N6	−0.001724 (81)†	N6*A*	0.000657 (47)†	N8	–	O81	0.000787 (60)†	O82	0.000494 (66)
*C*_222_	N6	0.000264 (49)	N6*A*	–	N8	−0.001359 (69)†	O81	0.000246 (63)	O82	0.000379 (95)
*C*_333_	N6	–	N6*A*	–	N8	−0.000232 (10)†	O81	−0.000044 (9)	O82	−0.000034 (8)
*C*_112_	N6	0.002705 (157)†	N6*A*	−0.000538 (93)	N8	−0.000386 (91)	O81	0.000655 (138)	O82	−0.000725 (165)
*C*_122_	N6	−0.001220 (128)	N6*A*	0.000432 (92)	N8	0.000902 (119)	O81	0.000411 (141)	O82	0.000856 (192)
*C*_113_	N6	–	N6*A*	0.000268 (51)	N8	−0.000303 (50)	O81	0.000324 (71)	O82	–
*C*_133_	N6	–	N6*A*	–	N8	0.000295 (34)	O81	–	O82	–
*C*_223_	N6	–	N6*A*	–	N8	−0.002084 (85)†	O81	–	O82	−0.000378 (101)
*C*_233_	N6	–	N6*A*	–	N8	−0.001178 (45)†	O81	–	O82	0.000386 (44)
*C*_123_	N6	–	N6*A*	–	N8	0.000991 (102)	O81	−0.000511 (114)	O82	–
										
70 K										
*C*_111_	N6	−0.000475 (56)	N6*A*	0.000135 (39)	N8	0.000313 (41)	O81	0.000466 (48)	O82	0.000519 (52)†
*C*_222_	N6	–	N6*A*	0.000422 (34)†	N8	–	O81	–	O82	–
*C*_333_	N6	–	N6*A*	−0.000061 (4)†	N8	–	O81	–	O82	–
*C*_112_	N6	−0.000874 (81)†	N6*A*	–	N8	0.000245 (58)	O81	−0.000604 (76)	O82	0.000770 (90)
*C*_122_	N6	−0.000247 (64)	N6*A*	–	N8	0.000321 (51)	O81	0.000308 (70)	O82	0.000713 (87)
*C*_113_	N6	–	N6*A*	–	N8	0.000175 (33)	O81	0.000348 (42)	O82	0.000237 (42)
*C*_133_	N6	–	N6*A*	0.000095 (17)	N8	0.000081 13)	O81	0.000100 (18)	O82	0.000064 (17)
*C*_223_	N6	–	N6*A*	−0.000627 (36)†	N8	0.000113 (23)	O81	0.000210 (33)	O82	–
*C*_233_	N6	–	N6A	0.000361 (18)†	N8	–	O81	−0.000084 (16)	O82	−0.000136 (17)
*C*_123_	N6	–	N6*A*	−0.000435 (51)	N8	0.000123 (38)	O81	–	O82	–

† 10σ level.

**Table 3:** Summary of the three strongest nitro group interactions at different temperatures

Cp	*T*(K)	Involved atoms	*D*12 (Å)	*D*1cp (Å)	*D*2cp (Å)	ρ_tot_ (e Å^−3^)	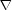 ^2^ρ (e Å^−5^)	λ_1_ (e Å^−5^)	λ_2_ (e Å^−5^)	λ_3_ (e Å^−5^)	ε	*G*(**r**_CP_) (kJ mol^−1^ a.u.^−3^)	*V*(**r**_CP_) (kJ mol^−1^ a.u.^−3^)	*H*(**r**_CP_) (kJ mol^−1^ a.u.^−3^)
Cp1	10	O81*A*—H62*A*	2.0316	1.281	0.751	0.109	2.14	−0.45	−0.45	3.05	0.00	46.7	−35	11.7
	35		2.0163	1.291	0.726	0.087	2.45	−0.36	−0.36	3.17	0.01	49.8	−32.9	16.9
	70		2.0393	1.302	0.737	0.086	2.34	−0.37	−0.37	3.08	0.01	47.8	−31.8	16.0
	100		2.0261	1.314	0.715	0.060	2.47	−0.25	−0.24	2.96	0.04	47.8	−28.1	19.7
Cp2	10	O82—H62	2.2634	1.408	0.886	0.060	1.21	−0.24	−0.22	1.66	0.09	24.8	−16.8	8.0
	35		2.2496	1.406	0.897	0.055	1.28	−0.25	−0.20	1.73	0.18	25.7	−16.6	9.1
	70		2.2930	1.418	0.912	0.053	1.14	−0.22	−0.20	1.56	0.08	23.1	−15	8.1
	100		2.3014	1.436	0.924	0.046	1.07	−0.21	−0.17	1.44	0.18	21.2	−13.4	7.8
Cp3	10	O81—H4*A*	2.3450	1.363	1.002	0.076	1.13	−0.26	−0.25	1.64	0.04	24.8	−18.8	6.0
	35		2.3489	1.375	1.005	0.069	1.09	−0.25	−0.24	1.58	0.03	23.5	−17.2	6.3
	70		2.3812	1.395	1.010	0.065	1.07	−0.25	−0.24	1.55	0.03	22.6	−16.2	6.4
	100		2.3671	1.396	1.001	0.058	1.05	−0.25	−0.21	1.50	0.15	21.7	−15.0	6.7

**Table 4:** Unit-cell parameters of **1** at different temperatures

		10 K	35 K	70 K	100 K
Single-crystal measurement	*a* (Å)	11.0104 (3)	10.9784 (14)	11.0470 (12)	11.030 (2)
	*b* (Å)	10.0398 (2)	10.0056 (13)	10.1293 (11)	10.092 (2)
	*c* (Å)	18.6040 (4)	18.488 (3)	18.652 (2)	18.637 (3)
	β (°)	97.320 (2)	97.223 (4)	97.223 (3)	97.24 (2)
Powder diffraction	*a* (Å)	–	11.0595	11.0491	11.0532
	*b* (Å)		10.1355	10.1156	10.1303
	*c* (Å)		18.6883	18.6742	18.6769
	β (°)		97.191	97.223	97.175

**Table 5:** Comparison of the C—C bond distances of the aryl ring for 35, 70 and 100 K data, using the cell parameters transferred from the powder experiment

*T* (K)	Atom 1	Atom 2	D12 (Å)	*T* (K)	Atom 1	Atom 2	D12 (Å)
35	C1	C2	1.405	35	C1*A*	C2*A*	1.401
70			1.400	70			1.398
100			1.398	100			1.396
35	C1	C6	1.413	35	C1*A*	C6*A*	1.412
70			1.410	70			1.409
100			1.409	100			1.407
35	C2	C3	1.401	35	C2*A*	C3*A*	1.396
70			1.396	70			1.393
100			1.395	100			1.392
35	C3	C4	1.404	35	C3*A*	C4*A*	1.406
70			1.401	70			1.403
100			1.400	100			1.401
35	C4	C5	1.400	35	C4*A*	C5*A*	1.396
70			1.394	70			1.392
100			1.392	100			1.390
35	C5	C6	1.418	35	C5*A*	C6*A*	1.415
70			1.417	70			1.414
100			1.415	100			1.412
