# Supplementary material for: Anharmonicity and isomorphic phase transition: a multi-temperature X-ray single-crystal and powder diffraction study of 1-(2′-aminophenyl)-2-methyl-4-nitroimidazole
Source: IUCrJ. 2014 Feb 28;1(Pt 2):110–8. doi: 10.1107/S2052252514002838 (PMC4062092; doi:10.1107/S2052252514002838)
Supplement: Supplementary file 13 [file m-01-00110-sup13.pdf]

IUCrJ

Volume 1 (2014)

Supporting information for article:

Anharmonicity and isomorphic phase transition: a multi-temperature X-ray single-crystal and powder diffraction study of 1-(2′-aminophenyl)-2-methyl-4-nitroimidazole

Agnieszka Poulain, Emmanuel Wenger, Pierrick Durand, Katarzyna N. Jarzembska, Radosław Kamiński, Pierre Fertey, Maciej Kubicki and Claude Lecomte

Supporting information

Anharmonicity and isomorphic phase transition: a multi-temperature X-ray single crystal and powder diffraction study of 1-(2'-aminophenyl)-2-methyl-4-nitroimidazole

Agnieszka Poulain,[a,b§] Emmanuel Wenger,[a] Pierrick Durand,[a]
Katarzyna N. Jarzembska,[c‡] Radosław Kamiński,[c‡] ,Pierre Fertey,[a]
Maciej Kubicki,[b]* Claude Lecomte[a]*

[a] CRM², Jean Barriol Institute, CNRS UMR 7036, University of Lorraine, BP 70239, Boulevard des Aiguillettes, 54506 Vandoeuvre-lès-Nancy, France

[b] Faculty of Chemistry, Adam Mickiewicz University, Umultowska 89B, 61-614 Poznań, Poland

[c] Department of Chemistry, University of Warsaw, Pasteura 1, 02-093 Warszawa, Poland

* Corresponding authors: Maciej Kubicki (mkubicki@amu.edu.pl),
Claude Lecomte (claude.lecomte@crm2.uhp-nancy.fr)

§ Current address: European Synchrotron Radiation Facility (ESRF), 6 Rue Jules Horowitz, BP 220, 38043 Grenoble Cedex 9, France

‡ Current address: Department of Chemistry, University at Buffalo, The State University of New York, Buffalo, NY 14260-3000, USA

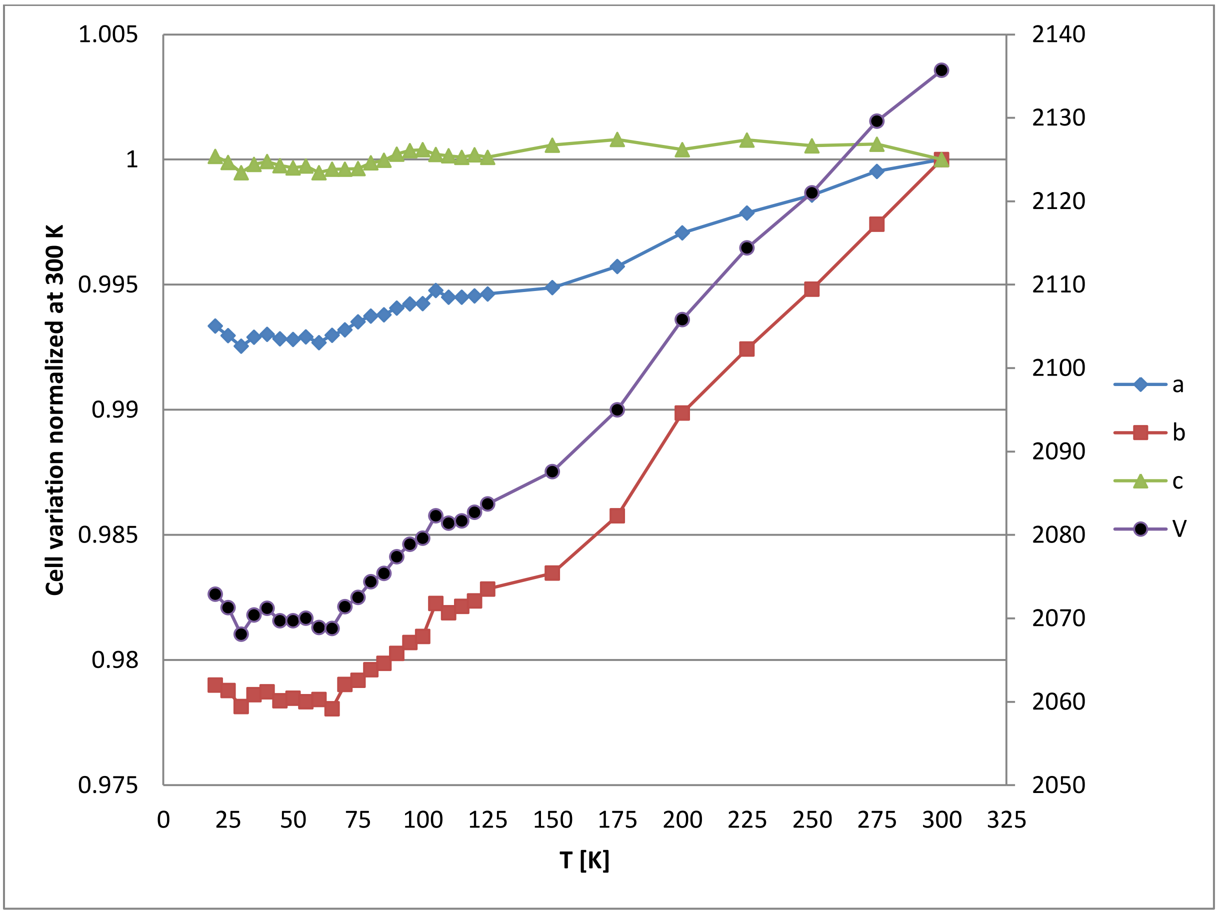

Figure S1 Unit cell parameters variation with temperature increase from 20 K to 300 K normalized at 300 K.

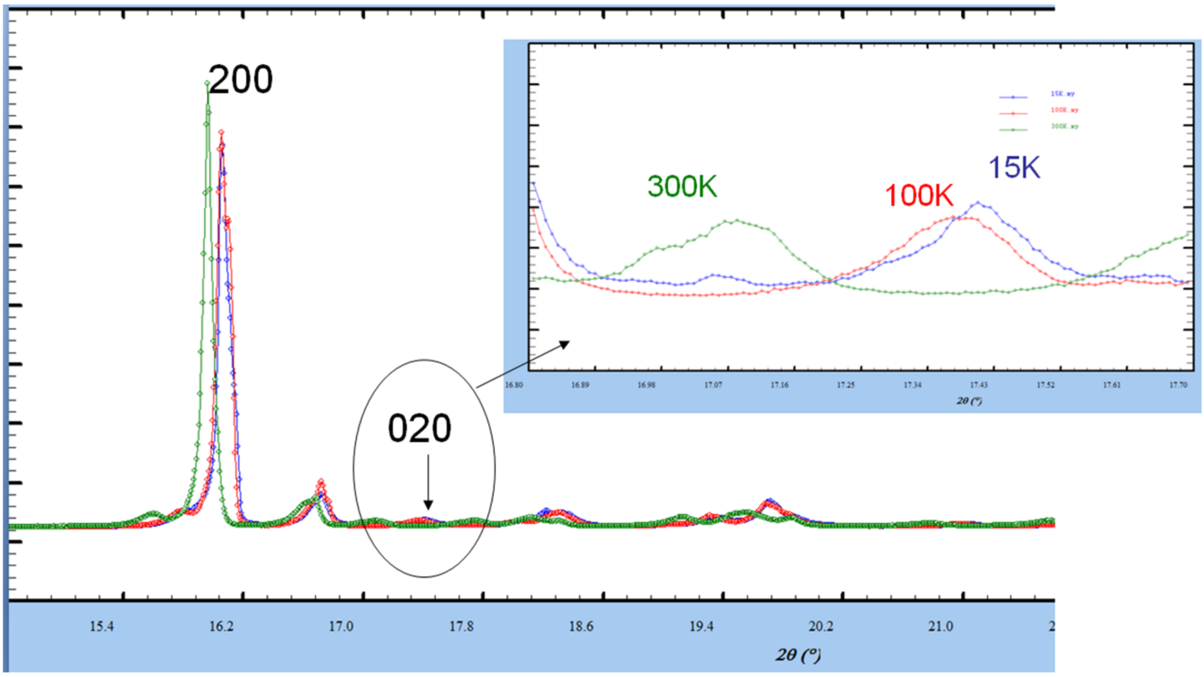

Figure S2 Position of 020 peak at different temperatures.

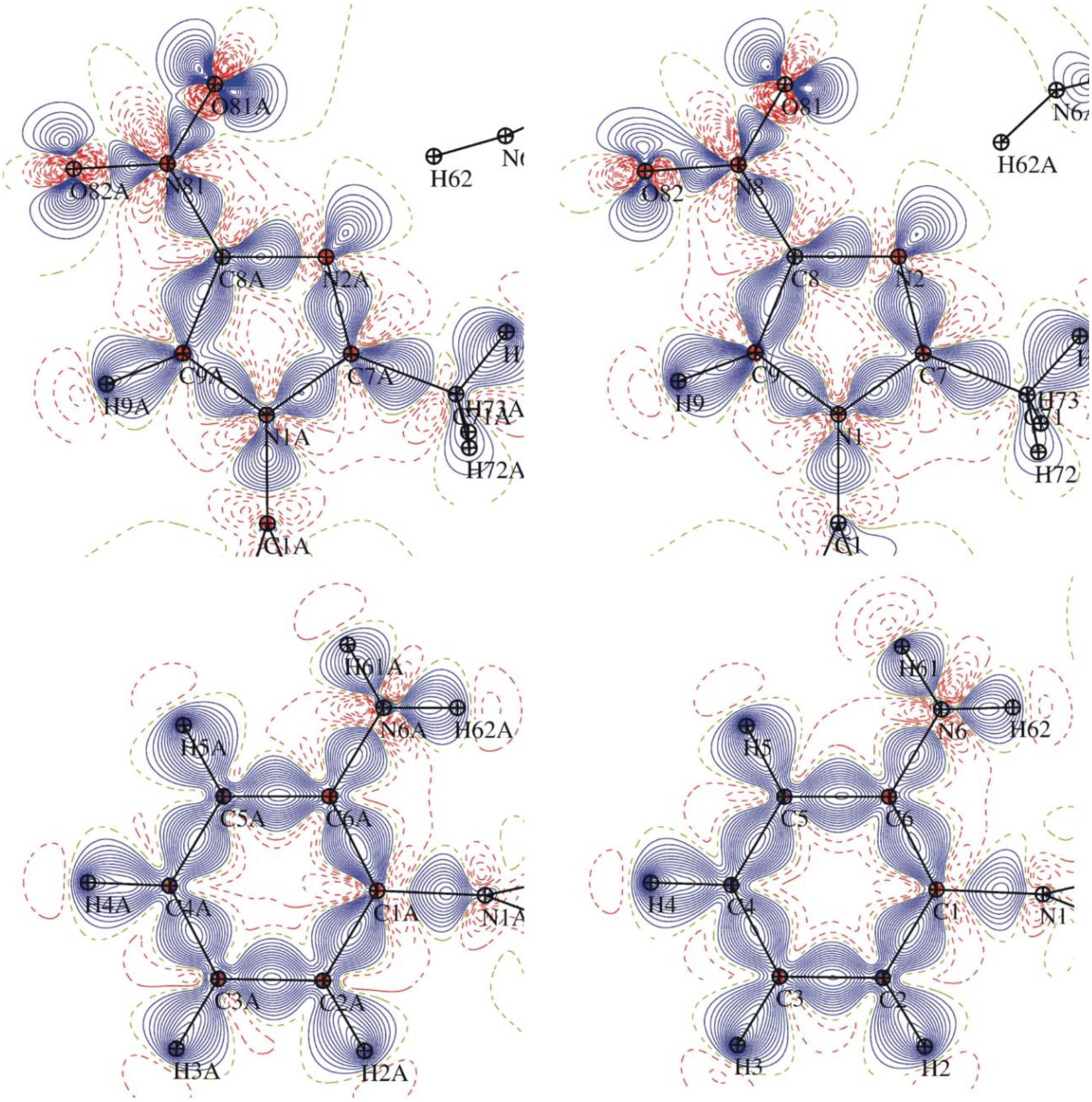

Figure S3 Static deformation electron density of **1** in the four main planes of the aromatic rings at 10 K after multipolar refinement (**harmonic**); contour 0.05 e/Å^3, blue positive, red negative.

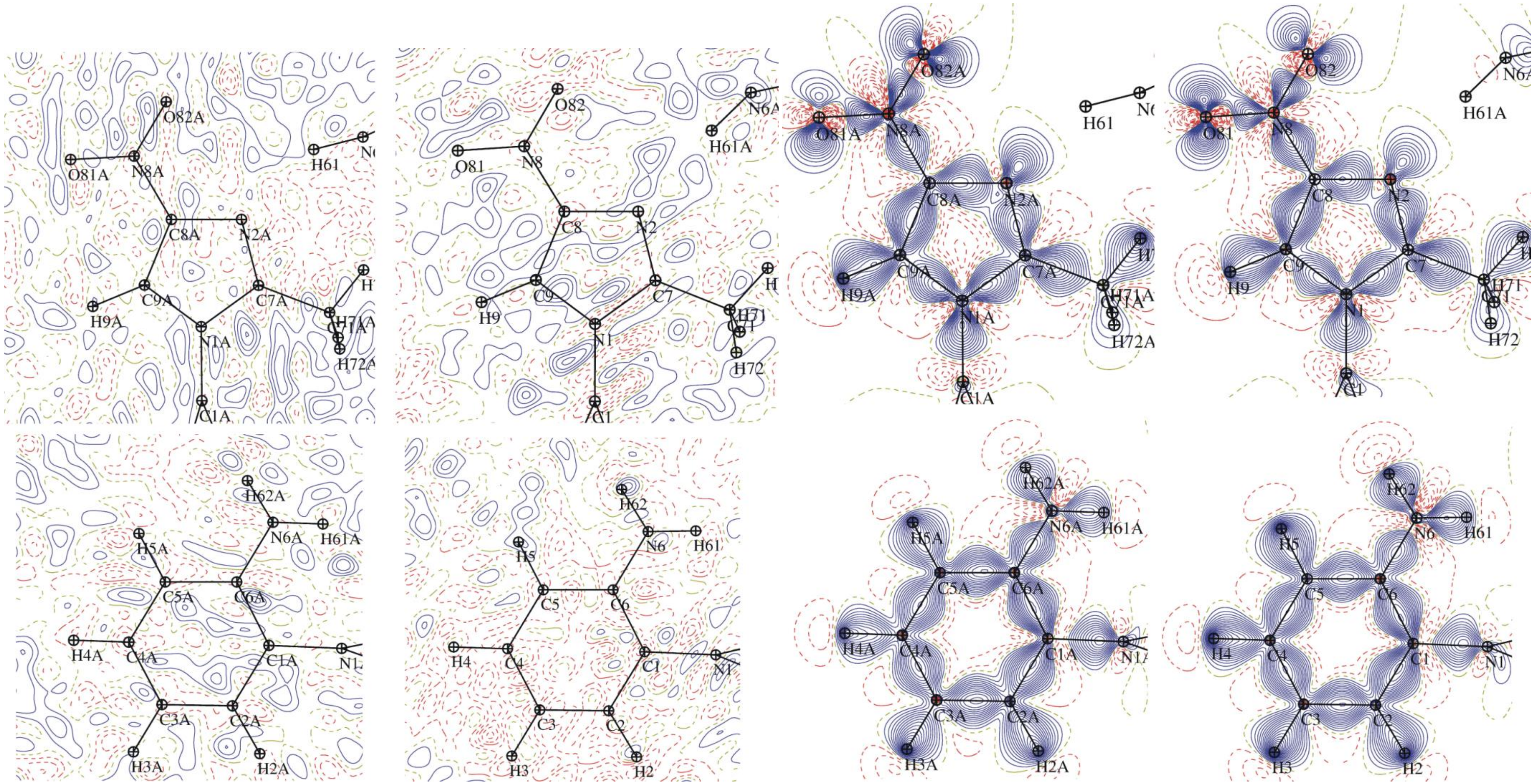

Figure S4 Residual (left) and static deformation density (right) maps of the electron density of **1** at 35 K drawn in the four main planes of the molecules after multipolar refinement (**harmonic**), contours 0.05e/Å3, blue negative, red positive, s < 0.9 $Å^{-1}$.

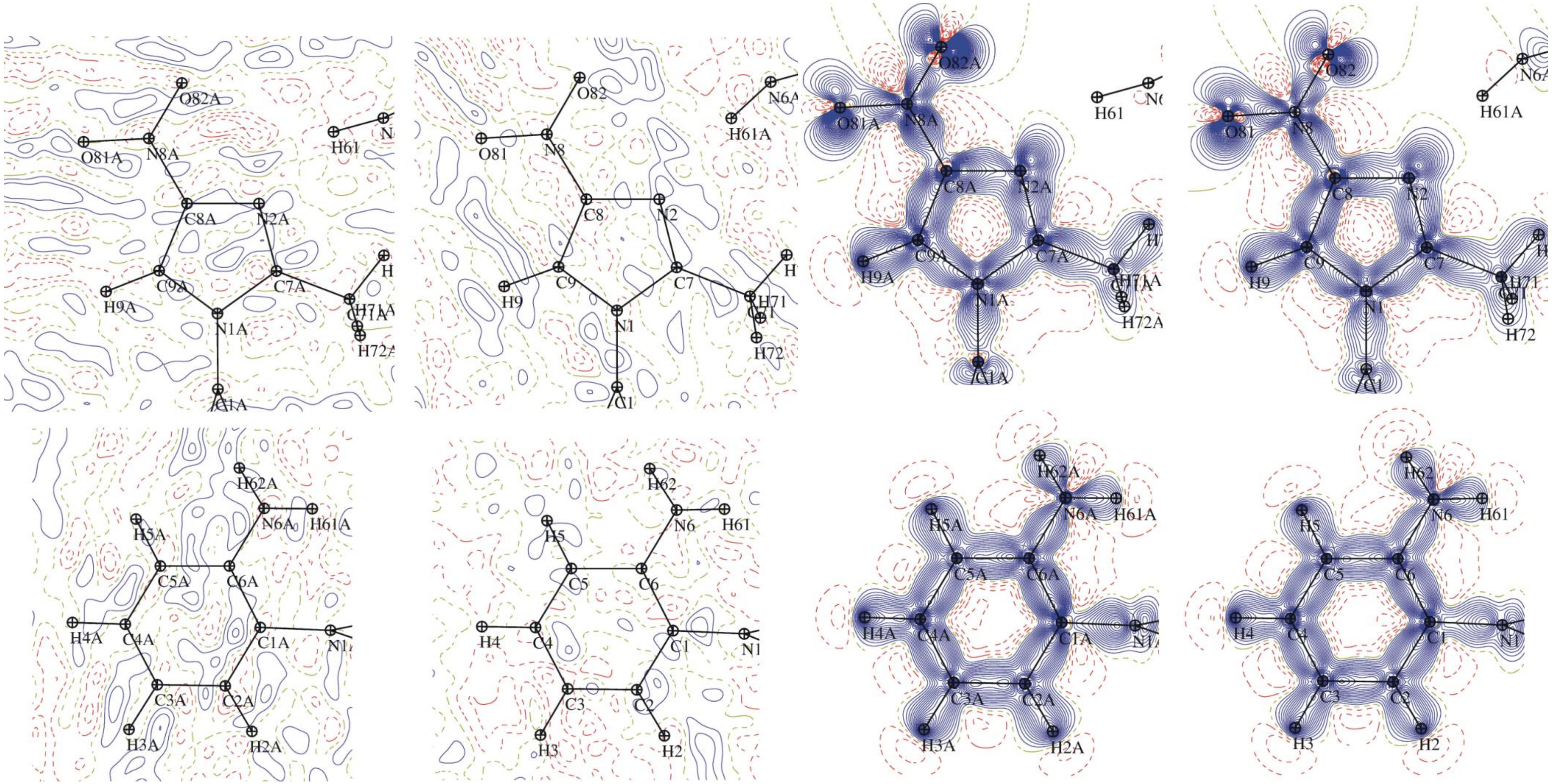

Figure S5 Residual (left) and static deformation density (right) maps of the electron density of **1** at 70 K drawn in the four main planes of the molecules after multipolar refinement with **anharmonic nuclear motion** for five problematic atoms, contours 0.05e/Å^3, blue negative, red positive, s < 0.9 Å^{-1}.

Table S1 Comparison of the characteristics of critical points for the anharmonic (at 70 and 100 K) fragment of the molecule

atoms of the phenyl ring											
T	atom 1	atom 2	D12 [Å]	D1cp [Å]	D2cp [Å]	ρ_{tot} [e/Å^3]	$\nabla^2\rho$ [e/Å^5]	λ_1 λ_2 λ_3 [e/Å^5]			ε
10	C1	C2	1.394	0.702	0.692	2.11	-19.7	-16.5	-12.7	9.6	0.23
35			1.388	0.716	0.672	2.20	-20.9	-17.3	-14.0	10.4	0.19
70			1.401	0.718	0.683	2.14	-16.4	-17.4	-13.7	14.7	0.21
100			1.393	0.695	0.698	2.21	-17.8	-17.7	-14.2	14.1	0.20
10	C1A	C2A	1.394	0.719	0.675	2.10	-19.5	-16.3	-12.7	9.5	0.22
35			1.387	0.711	0.676	2.15	-19.5	-16.7	-13.4	10.6	0.20
70			1.398	0.723	0.675	2.12	-15.8	-17.1	-13.4	14.8	0.21
100			1.393	0.704	0.689	2.18	-17.4	-17.3	-14.1	13.9	0.18
10	C1	C6	1.407	0.702	0.705	2.07	-18.1	-16.0	-12.5	10.4	0.22
35			1.397	0.720	0.678	2.17	-20.2	-17.3	-13.5	10.7	0.22
70			1.409	0.706	0.703	2.13	-16.0	-17.6	-13.8	15.4	0.21
100			1.405	0.706	0.700	2.15	-16.4	-17.0	-13.8	14.5	0.19
10	C1A	C6A	1.404	0.717	0.688	2.10	-19.7	-16.7	-12.9	9.8	0.23
35			1.397	0.702	0.695	2.19	-20.4	-17.7	-13.8	11.1	0.22
70			1.409	0.717	0.692	2.17	-17.3	-18.5	-14.3	15.5	0.22
100			1.403	0.712	0.691	2.17	-17.5	-17.6	-14.0	14.1	0.20
10	C2	C3	1.390	0.698	0.693	2.11	-20.1	-16.2	-13.0	9.1	0.20
35			1.385	0.704	0.680	2.17	-20.8	-16.6	-13.6	9.4	0.18
70			1.396	0.683	0.713	2.12	-16.7	-16.9	-13.8	14.0	0.18
100			1.391	0.713	0.678	2.18	-18.1	-16.8	-14.1	12.8	0.17
10	C2A	C3A	1.389	0.708	0.682	2.12	-20.6	-15.4	-13.2	7.9	0.14
35			1.385	0.708	0.677	2.13	-19.7	-15.6	-13.4	9.3	0.14
70			1.392	0.695	0.697	2.13	-16.8	-16.4	-13.8	13.5	0.16
100			1.389	0.720	0.669	2.18	-18.1	-16.2	-14.1	12.3	0.13
10	C3	C4	1.397	0.699	0.698	2.10	-19.7	-16.1	-13.1	9.6	0.18
35			1.389	0.682	0.706	2.16	-20.8	-16.8	-13.5	9.5	0.19
70			1.399	0.703	0.697	2.16	-17.6	-17.5	-14.4	14.4	0.18
100			1.397	0.688	0.709	2.17	-17.6	-16.7	-14.0	13.1	0.17
10	C3A	C4A	1.398	0.695	0.702	2.07	-19.7	-15.5	-12.6	8.4	0.18
35			1.391	0.688	0.703	2.11	-19.8	-15.7	-13.3	9.2	0.15
70			1.402	0.698	0.704	2.14	-17.6	-17.2	-14.2	13.8	0.18
100			1.397	0.677	0.720	2.16	-18.0	-16.4	-13.9	12.4	0.15
10	C4	C5	1.388	0.701	0.687	2.14	-20.5	-16.7	-13.0	9.2	0.22
35			1.383	0.700	0.683	2.17	-20.8	-16.9	-13.5	9.6	0.20
70			1.395	0.699	0.697	2.13	-17.1	-17.1	-14.1	14.1	0.18
100			1.387	0.709	0.678	2.20	-18.2	-17.4	-14.3	13.4	0.18
10	C4A	C5A	1.388	0.692	0.696	2.10	-20.0	-16.0	-12.6	8.7	0.21
35			1.381	0.697	0.684	2.13	-20.0	-16.0	-13.1	9.0	0.18
70			1.392	0.710	0.682	2.14	-17.6	-17.1	-14.0	13.5	0.18
100			1.387	0.713	0.674	2.19	-18.5	-17.1	-14.2	12.8	0.17
10	C5	C6	1.410	0.692	0.718	2.06	-18.6	-15.6	-12.6	9.7	0.19
35			1.402	0.696	0.706	2.09	-18.9	-16.2	-12.8	10.1	0.21

70			1.417	0.702	0.715	2.05	-14.8	-15.9	-13.4	14.5	0.16
100			1.410	0.691	0.720	2.06	-15.3	-15.9	-13.1	13.7	0.18
10	C5A	C6A	1.409	0.693	0.716	2.08	-19.3	-16.0	-12.5	9.2	0.22
35			1.405	0.675	0.730	2.10	-19.4	-16.2	-13.0	9.8	0.20
70			1.414	0.686	0.728	2.06	-15.6	-16.4	-13.3	14.0	0.19
100			1.409	0.681	0.728	2.06	-16.0	-15.9	-13.2	13.0	0.17
atoms with harmonic (10 and 35 K) and anharmonic (70 and 100 K) treatment											
T	atom 1	atom 2	D12 [Å]	D1cp [Å]	D2cp [Å]	ρ_{tot} [e/Å^3]	$\nabla^2\rho$ [e/Å^5]	λ_1 λ_2 λ_3 [e/Å^5]			ε
10	C6	N6	1.373	0.582	0.791	2.15	-20.6	-16.8	-15.1	11.3	0.10
35			1.368	0.587	0.782	2.19	-20.6	-17.5	-15.0	12.0	0.14
70			1.379	0.608	0.771	2.19	-15.3	-18.0	-15.5	18.1	0.14
100			1.372	0.604	0.769	2.25	-16.7	-18.5	-15.7	17.6	0.15
10	C6A	N6A	1.377	0.595	0.783	2.17	-20.6	-17.5	-14.8	11.7	0.15
35			1.372	0.593	0.779	2.24	-21.5	-18.6	-15.7	12.8	0.16
70			1.381	0.611	0.771	2.26	-17.1	-19.5	-15.6	18.0	0.20
100			1.374	0.615	0.759	2.27	-17.5	-19.0	-15.7	17.2	0.17
10	C8	N8	1.425	0.560	0.865	1.91	-20.0	-16.4	-12.2	8.5	0.26
35			1.415	0.566	0.849	2.05	-21.0	-17.6	-13.8	10.4	0.22
70			1.431	0.583	0.848	1.95	-14.1	-16.9	-13.5	16.3	0.20
100			1.424	0.583	0.841	1.98	-15.2	-16.9	-13.2	14.9	0.22
10	N8	O81	1.233	0.593	0.640	3.28	-10.5	-30.8	-27.8	48.0	0.10
35			1.228	0.599	0.629	3.28	-8.7	-30.0	-27.8	49.1	0.07
70			1.236	0.592	0.644	3.42	-11.4	-32.1	-30.0	50.7	0.06
100			1.232	0.594	0.638	3.48	-11.2	-32.3	-30.3	51.4	0.06
10	N8	O82	1.231	0.602	0.629	3.27	-12.3	-30.5	-28.0	46.1	0.08
35			1.228	0.600	0.628	3.39	-12.8	-32.4	-29.4	49.0	0.09
70			1.234	0.601	0.634	3.30	-8.8	-30.3	-28.1	49.6	0.07
100			1.227	0.583	0.644	3.44	-12.0	-32.6	-30.1	50.7	0.07
